# Accuracy of cardiac output estimations by transthoracic echocardiography compared with an accepted method of thermodilution, the pulmonary artery catheter, in the critically ill patients

**DOI:** 10.1186/2197-425X-3-S1-A598

**Published:** 2015-10-01

**Authors:** J Leache Irigoyen, J Marín Corral, I Oliva Zelaya, G Moreno Muñoz, V Blázquez Alcaide, M Bodí Saera, C Villavicencio Lujan

**Affiliations:** Intensive Care Medicine, Joan XXIII Tarragona University Hospital, Tarragona, Spain

## Objectives

To evaluate the accuracy of cardiac output (CO) measurements by intensive care unit (ICU) physicians performing transthoracic echocardiography with pulsed-wave doppler ultrasound (PWD), compared with the pulmonary artery catheter (PAC).

## Methods

Prospective observational cohort study. A total of 42 consecutive critically ill patients who required hemodynamic monitoring with pulmonary artery catheter were enrolled over a period of 18 months. Demographic data, reason for admission, comorbidities, APACHE II, SOFA, need for vasoactive drugs and acceptability of the ultrasound measurements, were analyzed. In each patient, three PWD-CO estimations were obtained by three different observers with basic echocardiography training. The PWD-CO was obtained using the maximum of three left ventricular outflow tract (LVOT) diameter measurements and the mean of three LVOT velocity time integral measurements. The PAC-CO was obtained averaging the results of 3 thermodilutions. The mean of the three PWD-CO estimations and the PAC-CO were compared to evaluate the agreement between the two methods. Patients with no regular sinus rhythm, aortic valve disease or with impossibility to perform the echocardiographic measurements were excluded. The interobserver accuracy and the agreement between both methods were measured by the coefficient of intraclass correlation (CIC) and a Bland-Altman plotting (BAP).

## Results

From a total of 42 studied patients, 22 were excluded. In 14 patients (33%) it was impossible to perform the echocardiographic measurements. The average age was 67 years, 79% male. The average of the APACHE II and SOFA scores were 23 (± DE 8.41) and 7 (± DE 2.86). The most frequent diagnosis on admission to the ICU was septic shock (43%) and the majority of patients required mechanical ventilation (83%) and vasoactive drugs (79%). The interobserver agreement was considered good according to the CIC 0.7 (IC 95% 0.48-0.86; p < 0.001). The BAP demonstrated acceptable agreement with a mean of differences 0.64 litres/minute, standard deviation of 1.18 litres/minute and 95% limits of agreement of -1.73 to 3.01 litres/minute. When The CO was lower than 6 litres/minute, the agreement improved, with a mean of differences 0.13 litres/minute, standard deviation of 0.89 litres/minute and 95% limits of agreement of -1.64 to 1.90 litres/minute.

## Conclusions

In critically ill patients with adequate echocardiographic images, the PWD-CO estimation can be reproducible between observers with basic echocardiographic training and showed acceptable agreement with PAC-CO. In the group of patients with CO less than 6litres/minute the agreement is even better, demonstrating the PWD-CO estimation reliable and useful in this group of patients.

**Figure 1:**
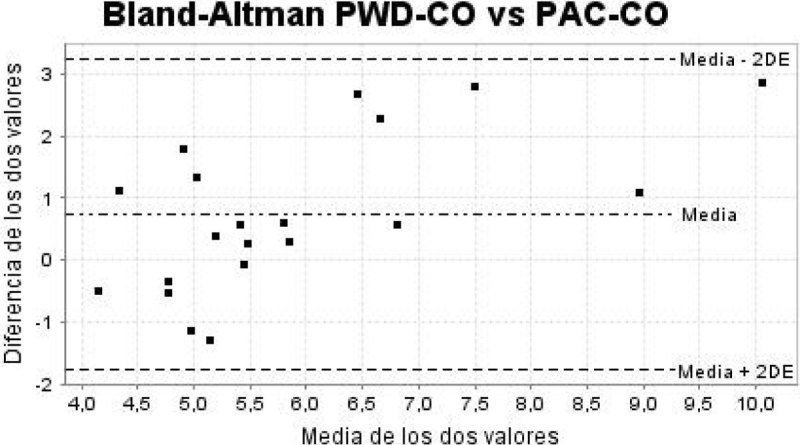
**Bland-Altman PWD-CO vs PAC-CO.**

**Figure 2:**
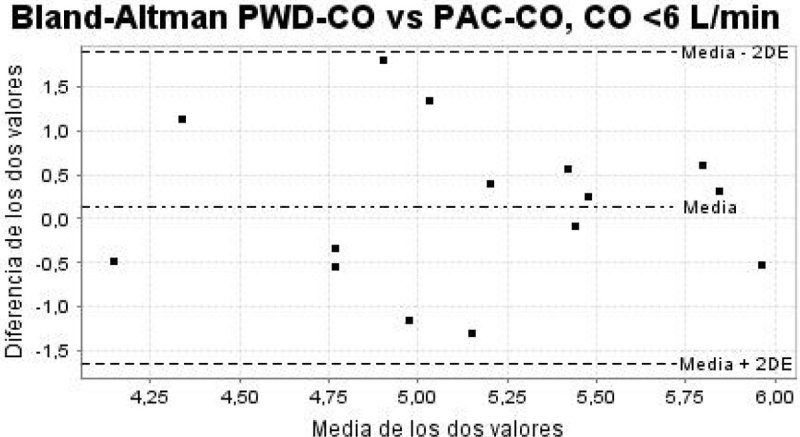
**Bland-Altman PWD-CO vs PAC-CO, CO <6 L/min.**

## References

[1] McLean AS, Needham A, Stewart D, Parkins R (1997). Estimation of cardiacoutput by non-invasive echocardiographic techniques in the critically illsubject. Anaesth Intensive Care.

